# Optimal Reactive Power Dispatch in ADNs using DRL and the Impact of Its Various Settings and Environmental Changes

**DOI:** 10.3390/s23167216

**Published:** 2023-08-17

**Authors:** Tassneem Zamzam, Khaled Shaban, Ahmed Massoud

**Affiliations:** 1 Electrical Engineering Department, Qatar University, Doha 2713, Qatar; 2 Computer Science and Engineering Department, Qatar University, Doha 2713, Qatar

**Keywords:** active distribution network, optimal reactive power dispatch, deep reinforcement learning, reward functions, hyperparameters, neural network, power loss, reactive power

## Abstract

Modern active distribution networks (ADNs) witness increasing complexities that require efforts in control practices, including optimal reactive power dispatch (ORPD). Deep reinforcement learning (DRL) is proposed to manage the network’s reactive power by coordinating different resources, including distributed energy resources, to enhance performance. However, there is a lack of studies examining DRL elements’ performance sensitivity. To this end, in this paper we examine the impact of various DRL reward representations and hyperparameters on the agent’s learning performance when solving the ORPD problem for ADNs. We assess the agent’s performance regarding accuracy and training time metrics, as well as critic estimate measures. Furthermore, different environmental changes are examined to study the DRL model’s scalability by including other resources. Results show that compared to other representations, the complementary reward function exhibits improved performance in terms of power loss minimization and convergence time by 10–15% and 14–18%, respectively. Also, adequate agent performance is observed to be neighboring the best-suited value of each hyperparameter for the studied problem. In addition, scalability analysis depicts that increasing the number of possible action combinations in the action space by approximately nine times results in 1.7 times increase in the training time.

## 1. Introduction

Optimal reactive power dispatch (ORPD) is a complex and vital problem in power systems planning and operation. It is a combinatorial optimization problem with nonlinearity. In active distribution networks (ADNs), load demand variations alter the required reactive power supply and hence cause load voltage variations. Through appropriate reactive power management, the adjustments of voltages can be achieved. A solution to ORPD minimizes distribution network power loss and improves its voltage profile while satisfying operational and physical constraints [1]. To accomplish its objectives, the ORPD solver obtains the optimal solution for the control settings of the utilized resources, such as utility-owned assets, namely capacitor banks (CBs), load tap changers (LTC), and voltage regulators (VR). It can also utilize inverter-interface resources, including those for distributed energy resources. Over the last decade, ORPD has gained increased attention to ensure the safe and secure operation of ADNs [2,3,4,5].

Diverse conventional optimization approaches have been employed to solve the ORPD problem, among them are Quadratic Programming [4], Interior-Point methods [6], Newton Raphson methods [3], and Nonlinear Programming [5]. However, these approaches have high computation time when handling complex objective functions with nonlinear characteristics and suffer from a lack of flexibility with real-world cases. To alleviate the aforementioned limitations, several approaches have been introduced, such as the Differential Algorithm [2,7,8], the Harmony Search Algorithm [9], the Grey Wolf Optimizer [1], Genetic Algorithms [10], Simulated Annealing [11], the Gravitational Search Algorithm [1], Particle Swarm Optimization [11,12], and the Artificial Bee Colony Algorithm [13]. However, these approaches greatly rely on precise optimization models. In other words, the existing model-based approaches are ineffective in the absence of a complete and accurate mathematical model of the distribution network. More efficient and intelligent solutions, such as machine learning approaches, especially reinforcement learning (RL) [14,15,16], have been proposed.

Derived by stimulus and response, RL is capable of learning from interactions with the environment rather than requiring complex mathematical models [15]. RL has become increasingly popular due to its achievements in addressing challenging sequential decision-making problems [17,18]. Combining RL with deep learning coins deep reinforcement learning (DRL), which achieved significant successes in several applications, such as AlphaGo, ATARI games, and robotics. These successes have motivated the use of DRL to solve different optimal control problems [15]. After adequate off-line training, the DRL agent can make a series of optimal decisions to achieve the best goal. Many published works have recently addressed DRL applications in power systems. These applications cover a wide range of decision, control, and optimization problems, including energy management [19,20,21,22], demand response [23,24,25,26], the electricity market [27,28,29,30], operational control [31,32,33,34], cybersecurity [35,36,37], economic dispatch [38,39], system optimization [40], edge computing [41], energy routing [42], and many others. 

To solve the ORPD problem, limited studies exist that propose different RL approaches. The work in paper [43] proposed a fully distributed Q-learning multiagent RL to minimize the active power loss while satisfying operational constraints. The authors in paper [44] developed a hierarchically correlated equilibrium Q-learning for reactive power optimization while considering carbon emission on the grid side. A decentralized multiagent DRL framework that utilizes reactive power control of photovoltaics (PVs), and storage systems was proposed in paper [45]. Other RL reactive power optimizations were presented in papers [46,47]. Nevertheless, there is a lack of literature addressing various challenges, such as adequate formulation of the reward function and finding the optimal combination of hyperparameters that effectively leverage network architectures’ strengths and training procedures to maximize performance. 

**Figure 1:**
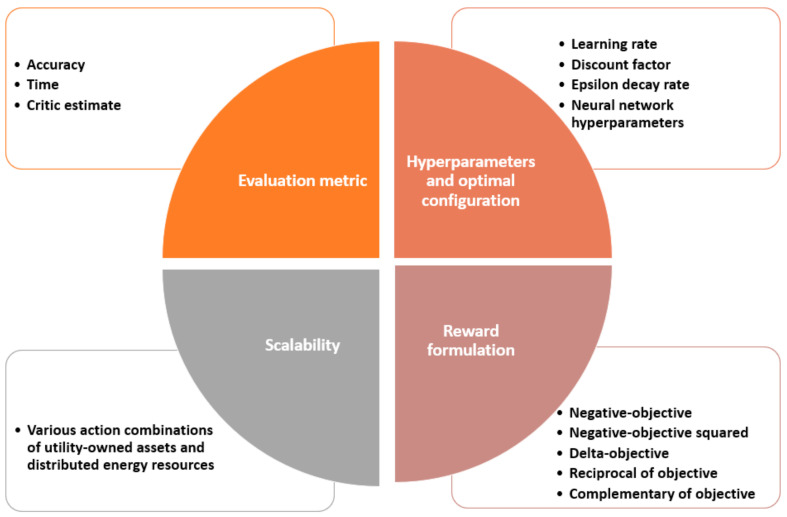
Core values of the presented work.

This paper examines the impact of DRL reward representations and hyperparameters on the agent’s learning performance when solving the ORPD problem for ADNs. We assess the agent’s performance in regard to accuracy and training time metrics, as well as critic estimate measures. Furthermore, different environmental changes are examined to study the scalability of the DRL model. To the best of our knowledge, no published work has addressed all these aspects of the proposed solution, i.e., DRL for ORPD in ADNs. The work in papers [48,49,50] focused on analyzing the reward shaping adversity. Different reward formulations for point-goal navigation and bidding applications are assessed in papers [48] and [49], respectively. In addition, to reward function analysis, the authors of paper [50] exploited different environments observing the accuracy and time of convergence. On the other hand, the work in papers [51,52] investigated RL hyperparameters. The authors in paper [51] employed a robot confrontation and a maze walker problem and studied the influence of the learning rate hyperparameter. They also demonstrated the average rewards and convergence of such problems. While in paper [52], the authors discussed the effect of the discount factor and the number of neurons of the neural network (NN) hyperparameters for the navigation of unmanned aerial vehicles as mobile base stations for long-term communication coverage. They also examined the performance of the RL agent for different numbers of these vehicles. Table 1 summarizes the main differences between the analysis performed in this paper and the recent RL-related analysis in other domains, and Figure 1 addresses the core values of the presented work. From Table 1, it can be observed that research works either focused on studying the effect of reward shaping on the agent performance or examining the hyperparameters of their problem with respect to the commonly used agent evaluation metric, namely, its accuracy. Although the agent’s accuracy in achieving its objectives is essential, it is crucial to highlight that examining the critic estimate, which resembles the long-term future reward and its progression throughout the training process, significantly impacts the agent’s performance. It is worth noting that the analysis of the critic estimate has not been studied before in works that assess RL agent performance in the power system field or other fields.

This paper focuses on the impact of several DRL elements on the agent’s learning performance through solving the ORPD problem. Specifically, we analyze the influence of different reward representations, hyperparameters, and a number of incorporated assets. The deep Q-Network (DQN) algorithm is used to solve the ORPD at medium voltage distribution grids by coordinating the reactive power output of different distributed energy resources, such as PVs, wind turbines, and battery storage, as well as traditional assets, such as CBs and LTCs. The main contributions of this paper are summarized in the following:

- A thorough study of the impact of various RL reward function representations on the agent learning performance. Specifically, squaring, reciprocal, and complementary reward function formulations, along with traditional techniques, are investigated. Furthermore, different environmental changes are examined to study the DRL model’s scalability through varying the number of incorporated assets.
- A detailed analysis of the effects on RL learning performance of different hyperparameters, including learning rates, discount factors, epsilon decay rates, and other NN-related hyperparameters. The analysis concludes with a set of optimal combinations of hyperparameters that can serve as a starting point for similar problems. Furthermore, we examine the critic estimate of the RL agent for the studied hyperparameters.

The rest of the paper is structured as follows. A brief background on RL is presented in Section 2. The ORPD RL-based problem is formulated and modeled in Section 3. ORPD DRL-based performance analysis is presented in Section 4. Finally, the work is concluded in Section 5.

## 2. Reinforcement Learning (RL) and Deep RL (DRL)

RL is formulated as a Markov Decision Process (MDP) defined by a 5-tuple $\left(S,A,P,R,\gamma \right)$, where

S is the set of states, ∀𝓈∈S; 

A is the set of actions, ∀𝒶∈A; 

$P\;:\;S\;x\;A\;x\;S\;\to \left[0,1\right]$ is the transition function defined by the probability that an action 𝒶 at time t is chosen, will transition the system at time t+1 from state 𝓈 to state ${𝓈}^{\prime }$, that is ${𝒫}_{a}\left(𝓈,{𝓈}^{\prime }\right)=𝒫({𝓈}_{t+1}={𝓈}^{\prime }\;|{𝓈}_{t}=𝓈,\;{𝒶}_{t}=𝒶)$; 

R : S x A x S → ℝ is the reward function, where $R_{a}\left(𝓈,{𝓈}^{\prime }\right)$ represents the immediate reward received by the agent after executing an action that transitioned the environment states from 𝓈 to 𝓈′; 

γ is the discount factor, where $\gamma =\left[0,1\right]$, which is the balance between the current rewards and the future rewards. 

An essential property of MDPs is the Markov property [14] which states that the state transitions depend only on the recent state and action of the system and are independent of the prior environment states and actions, that is $𝒫({𝓈}_{t+1}={𝓈}^{\prime }\;,{𝓇}_{t+1}=𝓇|\;{𝓈}_{t},\;{𝒶}_{t})$ for all ${𝓈}^{\prime },$ 𝓇, ${𝓈}_{t}$, and ${𝒶}_{t}$. In the real world, the transition probabilities P and the rewards R are unknown. Hence, RL represents an extension and generalization over MDPs. 

RL is a branch of machine learning motivated by behaviourist psychology, which examines the decision-making performance of artificial agents in an environment to reach a specific objective. The decision-making element in RL is the agent, and everything the agent interacts with is its environment. Specifically, the agent has to take actions sequentially to control a dynamic system, i.e., the environment. The environment is described by its dynamics, states, and a function that defines the state’s evolution based on the actions taken by the agent. The RL agent and environment interact in discrete time steps, i.e., t=0,1, 2, …. At each timestep, the agent receives the current state of the environment ${𝓈}_{t}\in S$ where S is the set of states and, based on it, selects an action ${𝒶}_{t}\in A,$ where A is the set of actions. After executing the action chosen by the agent, the environment moves to a new state ${𝓈}_{t+1}$ and the agent receives a scalar value, termed reward ${𝓇}_{t+1}$. The reward evaluates the correctness of the chosen action and, consequently, how far it is from the objective.

To achieve the defined objective, the agent has to learn a strategy, a.k.a. policy, to select the actions based on its interaction with the environment. Also, the states of a system define the required information that helps predict the evolution to the next state of the environment, given an executed action. Thus, the goal of an RL agent is to learn a policy. Then it selects the proper actions, given an observed current state of the environment, so that the expected sum of obtained rewards is maximized over time. The associated states, actions, and rewards differ from one RL problem to another. However, the framework remains the same. The continuous agent-environment interaction process makes decision-making behavior a step-by-step process. Most RL tasks can be decomposed into sequences between initial and terminal states of agent-environment interactions, where a sub-sequence is called an episode. Upon reaching a terminal state, the environment resets to the initial state.

**Figure 2:**
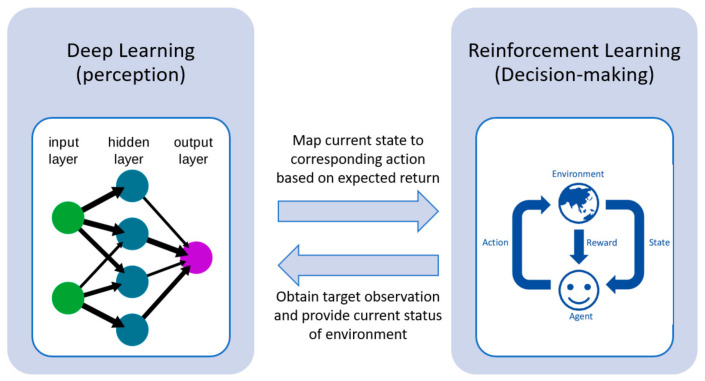
Deep reinforcement learning framework [33].

DRL integrates the perceived role of deep learning within the decision-making of RL. Figure 2 illustrates the framework of DRL. Deep learning obtains the target observation information from the environment and provides the state information in the current environment. The RL then maps the current state to the corresponding action and evaluates values based on the expected return [33]. The progress from RL to DRL has gone through a long development process. In classical tabular RL, e.g., the Q-learning approach, for small-scale state and action spaces, the representation of approximate value functions as arrays or tables is efficient. In this case, the methods can often find the exact optimal value functions and the optimal policies [43]. However, conventional RL approaches suffer from the ‘curse of dimensionality’ in real-world problems comprising of large-scale continuous action and/or state spaces. Deep NNs overcome this issue by providing compact low-dimensional representations of high-dimensional inputs [15], where the approximate value functions are represented as a parameterized functional form with a weight vector instead of a table. DRL can handle complicated tasks with lower prior knowledge due to its ability to learn levels of abstractions from data [40].

## 3. The Optimal Reactive Power Dispatch (ORPD) Case Study

The ORPD aims to minimize active power loss and improve the voltage profile of the power system while satisfying defined constraints. In this paper, the setting points of PV inverters in the ADN are coordinated to adjust the amount of reactive power supplied/absorbed to reduce the real power loss in transmission lines. Accordingly, the objective is to minimize active power loss while satisfying the standard voltage limits, as well as the reactive power limit of the PV inverters. Thus, the optimization problem is formulated as follows:

(1) $$\min\overset{nl}{\underset{k=1}{\sum }}P^{st}{}_{k,t}-P^{en}{}_{k,t}$$

s.t

(2) $$V_{min}\leq \left|V_{i,t}\right|\leq V_{max}$$

(3) $$\left|Q_{x,t}{}^{PV}\right|\leq \sqrt{{\left(S_{max}{}^{PV}\right)}^{2}-{\left(P_{x,t}{}^{PV}\right)}^{2}\;}$$

where 

nl denotes the number of branches in the network, 

$P^{st}{}_{k,t}$ and $P^{en}{}_{k,t}$ are the active power at the start and end of the $k_{th}$ branch at time t, respectively;

$V_{i,t}$ is the voltage of the $i^{th}$ bus at time t; 

$V_{min}$ and $V_{max}$ are the lower and upper voltage limits according to the ANSI standard, respectively; 

$Q_{x,t}{}^{PV}$ is the reactive power absorbed or supplied from the $x^{th}$ PV unit at time t;

$P_{x,t}{}^{PV}$ is the active power generation of the $x^{th}$ PV unit at time t and

$S_{max}{}^{PV}$ is the rating of the PV inverter. 

When designing an RL algorithm, an MDP model needs to be defined, which requires making several critical decisions, e.g., defining the state and action spaces, formulating the reward function, and selecting the RL agent that best suits the application. For the RL algorithm of ORPD for an ADN, the environment is the distribution network, and its dynamics are based on the AC power flow solver. Based on the objective, the reactive output power of PVs is to be coordinated to minimize power loss. Thus, the actions are the reactive power supplied/absorbed by PV. Discrete actions are considered; thus, the action space A becomes a set of all possible combinations of the reactive output power of PVs, i.e., from K to −K with a step size of 0.1. The selection of state variables is a crucial decision. Irrelevant states can cause the RL agent to suffer from the curse of dimensionality and increase training time. Contrarily, not considering vital state variables can lead to improper RL agent training and, consequently, inaccurate decisions. In the ORPD problem, the RL agent decides the amount of reactive output power of the PVs in the network based on the current demand and its generation. Thus, the required vital state variables by the RL agent are the active and reactive power demands and the PV’s active power generation. Accordingly, the state space is $S=\left[P\;Q\;PV\right]$, containing continuous variables. At time instant t, the state is ${𝓈}_{t}=\left[p_{t}\;q_{t}\;pv_{t}\right]$.

The reward function evaluates the agent’s performance due to the chosen action on the environment. Hence, the agent is reinforced with positive rewards if correct actions are chosen and if actions do not lead to a constraint violation, and reinforced with negative rewards if wrong actions that lead to constraint violation are taken. The objective of the ORPD is to minimize power loss; thus, the reward function $r_{1}$ is formulated as in (4), where p is the calculated power loss from the load flow solved at a step of the episode. In addition, to penalize the agent if a wrong action is taken, a total of three terms, $r_{2}$, $r_{3}$, and $r_{4}$, are added to the reward function. Since the voltage constraint is considered, $r_{2}$ accounts for violating the upper voltage limit and $r_{3}$ accounts for violating the lower voltage limit. Furthermore, to penalize for the violation of PV reactive output power constraint in (3), $r_{4}$ is considered. Consequently, the total reward function is given in (8).

(4) $$r_{1}=\left(1-p\right)$$

(5) $$r_{2}=\begin{array}{c} -\left(0.95-Vmin\right)\;\;\;\;\;\;\;\;\;if\;Vmin<0.95\\ 0\;\;\;\;\;\;\;\;\;\;\;\;\;\;\;\;\;\;\;\;\;\;\;\;\;\;\;\;\;\;\;\;\;otherwise \end{array}$$

(6) $$r_{3}=\begin{array}{c} -\left(Vmax-1.05\right)\;\;\;\;\;\;\;\;\;\;if\;Vmax>1.05\\ 0\;\;\;\;\;\;\;\;\;\;\;\;\;\;\;\;\;\;\;\;\;\;\;\;\;\;\;\;\;\;\;\;\;\;\;otherwise \end{array}$$

(7) $$r_{4}=\begin{array}{c} -\left[\left|Q\right|-\sqrt{S^{2}-PV^{2}}\right]\;\;\;\;\;\;\;\;\;\;if\;\left|Q\right|>\sqrt{S^{2}-PV^{2}}\\ 0\;\;\;\;\;\;\;\;\;\;\;\;\;\;\;\;\;\;\;\;\;\;\;\;\;\;\;\;\;\;\;\;\;\;\;otherwise \end{array}$$

(8) $$R_{t}=Nr_{1}+Mr_{2}+Mr_{3}+Gr_{4}$$

where

Vmin and Vmax are the minimum and maximum bus voltages in the network at a step in an episode, respectively, and

N, M, and G are constants to scale up the reward and penalty terms. 

The value of the constants N, M, and G are adjusted such that the resulting terms are in the same range. Accordingly, these constants depend on the examined network, demand, and PV generation.

**Figure 3:**
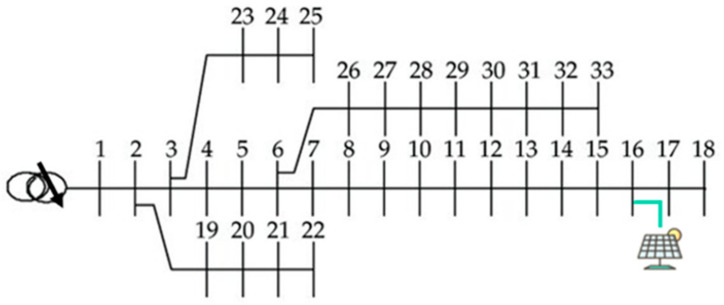
Modified IEEE 33-bus system.

In this paper, we solve the ORPD by a DRL with a deep Q-network (DQN) and analyze its performance. The ORPD-DQN-based algorithm is tested on a modified IEEE 33-bus system, as shown in Figure 3. A PV plant is installed at bus 16 with a 0.6 MVA inverter rating. The distribution network peak demand is 8.0 + 2.6 MVA, the substation voltage at bus 1 is 1.04 p.u (12.67 kV), and the minimum and maximum voltage limits are 0.95 p.u and 1.05 p.u, respectively. Table 2 lists the considered parameter settings of the system. Data of residential active power over one year from paper [53] is used as the training dataset. The RL algorithm is implemented on MATLAB using the Reinforcement Learning Toolbox [54], and the power flow calculation is based on MATPOWER 7.1 [55]. 

## 4. Performance Analysis of the ORPD DRL-based Solution

### 4.1. DRL Hyperparameters

The performance of the DRL agent highly depends on various parameters. The key sensitive parameters are the RL agent hyperparameters and the NN model parameters. Hyperparameters include the learning rate (LR), which controls the rate at which the NN learns the problem; the discount factor (DF), which manages the importance of future rewards over immediate rewards; and the є-greedy decay rate (EDR), which controls the balance of exploration and exploitation. NN parameters include the number of nodes in the hidden layers and the number of hidden layers in the model.

Generally, there is no known way to decide whether a higher or lower value of a specific parameter will improve the performance of the RL agent. Hence, to achieve the optimal suitable combination of parameters, multiple trial runs of training should be made to analyze the effect of each parameter on the performance of the RL agent for the specific problem. Other factors that affect the training performance are the training dataset, its size, and the method of reward function formulation. In other words, the parameters are problem-specific, and the best combination is mostly not intuitive. Due to this widely adopted manual tuning of parameters, it becomes a time-consuming process. In the following sections, the analysis of the effect of RL-agent hyper- and NN model parameters of the ORPD-DQN agent are examined by observing two main aspects: the episode and critic estimate (Q0) curves.

#### 4.1.1. The Episode Curves

The episode curve is analyzed by assessing the two performance indices: (1) the maximum reward obtained by the DQN agent ($R_{max}$), and (2) the episode number at which the maximum reward is attained by the DQN agent ($E_{R\_max}$). The ORPD-DQN agent is trained with different hyperparameters as follows:

- Learning rate (LR)

Deep NNs are trained using the stochastic gradient descent (SGD) method, which estimates the error gradient of the current state based on the training dataset and uses the back-propagation algorithm to update the model’s weights. LR represents the amount that the weights are updated each time. Typically, its value ranges from 0.0 to 1.0 and it is set during the creation of the critic function approximator of the NN. Generally, a large learning rate makes the neural network learn faster, however, at the cost of reaching a sub-optimal solution due to over-shooting. While given a small learning rate, the NN takes a longer time to learn to arrive at the optimal solution. Nevertheless, a small learning rate may never converge or get stuck in a sub-optimal solution, and a large learning rate will cause oscillations due to the large weight updates and diverge.

**Figure 4:**
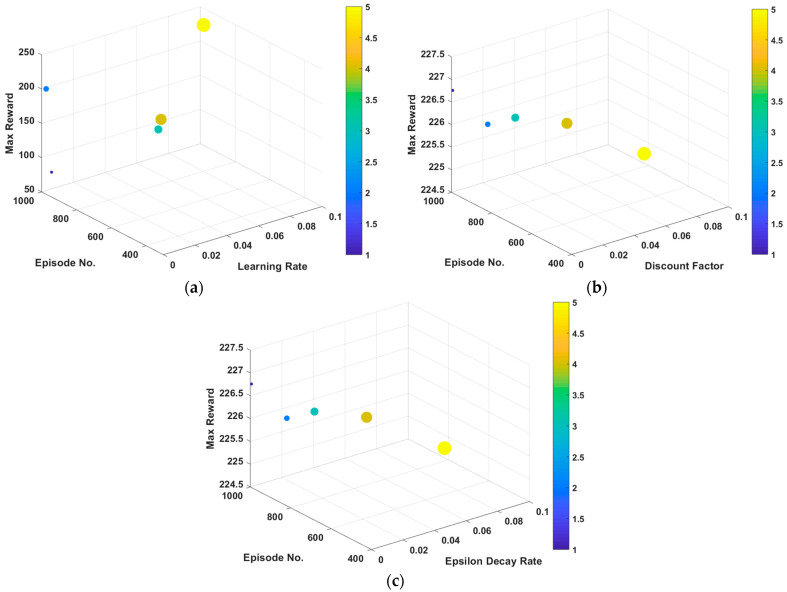
RL-agent hyperparameters analysis of maximum reward, $R_{max}$, and episode number, $E_{R\_max}$ concerning change in (**a**) learning rate; (**b**) discount factor; and (**c**) epsilon decay rate.

The performance of the DQN agent for ORPD is examined with different LRs. With other parameters fixed, $R_{max}$ and $E_{R\_max}$ are observed for LR values of 0.1, 0.01, 0.001, 0.0001, and 0.00001. Figure 4a demonstrates the maximum reward and the episode at which it is obtained for the studied values of the LR. It can be seen that as the learning rate decreases, the RL agent learns slower and reaches the highest reward in later episodes. However, near the optimal value of LR for the problem, the agent reaches its maximum reward faster. This indicates that the agent learnt and can adequately take actions that do not lead to penalization due to constraint violation much earlier. It can be induced that with an LR of 0.1, 0.0001, and 0.00001, the $E_{R\_max}$ is approximately 1000. On the other hand, LRs of 0.1 and 0.01 exhibit a much lower value of $E_{R\_max}$, around 400. Also, it can be realized that with an LR of 0.00001, the agent struggles at learning and gets stuck in a sub-optimal solution. This can be interpreted from the extremely low value of $R_{max}$, compared with the other values of LRs that show similar $R_{max}$ performance.

- Discount factor (DF)

The DF is a configurable parameter that prioritizes present and future rewards. Immediate rewards are essential since they can encourage and motivate the RL agent. Also, future rewards accompany higher risks due to uncertainty. Thus, they need to be discounted. On the other hand, giving greater importance to immediate rewards can lead the RL agent to get stuck at sub-optimal solutions. Typically, the DF value can range from 0, when caring only about immediate rewards, to 1.0, when caring only about future rewards, and is set when defining the options of the utilized agent.

For the DF analysis with respect to the ORPD-DQN agent performance, other parameters are fixed, and $R_{max}$ and $E_{R\_max}$ are observed for DF holding the common values of 0.8, 0.85, 0.9, 0.95, and 0.99. The maximum reward and its episode number, obtained for the studied values of DFs, are shown in Figure 4b. It can be noticed that with all DFs, the DQN agent obtains similar values of maximum reward, as indicated from the bars related to $R_{max}$. However, the values of $E_{R\_max}$ vary significantly from 500 to 1000. Also, it can be concluded from Figure 4b that as the DF increases, the DRL agent reaches the optimal solution faster, since it focuses more on future rewards. However, with a relatively high DF for the studied ORPD problem, a value of 0.99, the agent shows slower performance which can be translated from the increase of $E_{R\_max}$.

- Epsilon decay rate (EDR)

The balance between exploration and exploitation is managed through the configuration of the value of the EDR. Commonly, the default values of the starting value of epsilon and the minimum value of epsilon for the DQN algorithm are 0.9 and 0.1, respectively. Thus, the epsilon starts with a large value—where the agent randomly takes actions—and decays over time as training progresses to a small value where the agent takes actions based on the highest Q-values that are previously learnt. The amount with which the epsilon value decreases is known as the EDR and can be configured when defining the options of the utilized agent. Usually, it is set to a small value to ensure that the agent explores the state and action spaces before making decisions based on its experience.

Different EDRs are studied for the ORPD DQN-based agent performance through fixing other parameters and observing the $R_{max}$ and $E_{R\_max}$ for EDR holding values of 0.0005, 0.002, 0.005, 0.009, and 0.02. Figure 4c illustrates the maximum reward and the episode at which it is obtained for the studied values of EDRs. It can be observed from Figure 4c that as the EDR decreases, the RL agent stays longer in the exploration phase and takes more episodes to reach the optimal solutions, which is indicated from the decreasing trend of $E_{R\_max}$. However, increasing the EDR can increase the risk of the agent not having enough episodes learning the state and action spaces, leading to sub-optimal solutions. It can be elucidated from the obtained values of $R_{max}$ for the studied EDRs with an EDR of 0.0005, 0.002, 0.005, and 0.009, that the DRL agent achieves $R_{max}$ value of around 200, while with an EDR of 0.02, the agent attains a much lower reward of approximately 150.

- Neural network parameters

The two main hyperparameters of NNs configured to control the architecture of the network are the number of hidden layers and the number of nodes in each hidden layer. Each parameter requires a separate analysis to capture the values that best suit the specified ORPD problem and the training dataset. This paper defines a feed-forward with ReLU activation functions network for the critic network’s design. The performance of the DQN agent for ORPD is examined with different numbers of nodes and hidden layers of the NN.

- Number of neurons

**Figure 5:**
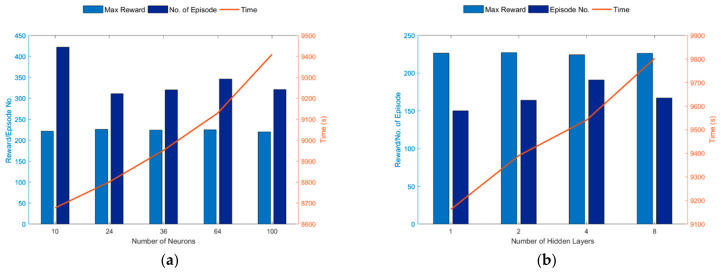
NN-hyperparameters analysis of maximum reward, $R_{max}$, episode number, $E_{R\_max}$, and training time concerning change in (**a**) the number of neurons in each layer of the NN; and **(b**) the number of NN hidden layers.

Figure 5a shows $R_{max}$ and $E_{R\_max}$ of the ORPD with different numbers of nodes 10, 24, 36, 64, and 100 in each hidden layer while fixing the other parameters. Generally, increasing the number of nodes in each hidden layer will enhance the training performance. However, it can also cause overfitting. It is essential to highlight that the degree of performance enhancement will considerably depend on the size of the training dataset. From Figure 5a, it can be realized that for the tested number of nodes on the studied ORPD problem, the agent achieved similar results in terms of $R_{max}$ and $E_{R\_max}$, with numbers of nodes of 10 and 100 having the weakest performances. Another factor that significantly affects the decision of the suitable value of the number of nodes hyperparameter for the application, and is vital to consider, is the training time. This is because the training time increases as the number of nodes in the neural network increases. Figure 5a also shows the effect of the number of nodes of the NN on $R_{max}$ and $E_{R\_max}$, while incorporating the influence of the training time.

- Number of hidden layers

$R_{max}$ and $E_{R\_max}$ analysis of the ORPD with respect to varying the number of hidden layers in the NN architecture is demonstrated in Figure 5b. Other parameters are fixed, and values of 1, 2, 4, and 8 of the number of hidden layers of the NN are examined. Increasing the number of hidden layers will enhance the training performance. However, it can also cause overfitting and prompt reverse performance. Similar to the impact of the number of nodes on the agent, the extent to which performance is enhanced by virtue of the number of hidden layers depends on the size of the training dataset. It can be deduced from Figure 5b that comparable results in terms of $R_{max}$ and $E_{R\_max}$ are realized by the agent for the tested number of hidden layers on the studied ORPD problem. Since the NN hyperparameters are associated with a direct correlation with respect to training time, Figure 5b is generated to realize the effect of the number of hidden layers of the NN on $R_{max}$ and $E_{R\_max}$, while incorporating the influence of the training time.

#### 4.1.2. The Critic Estimate (Q0)

**Figure 6:**
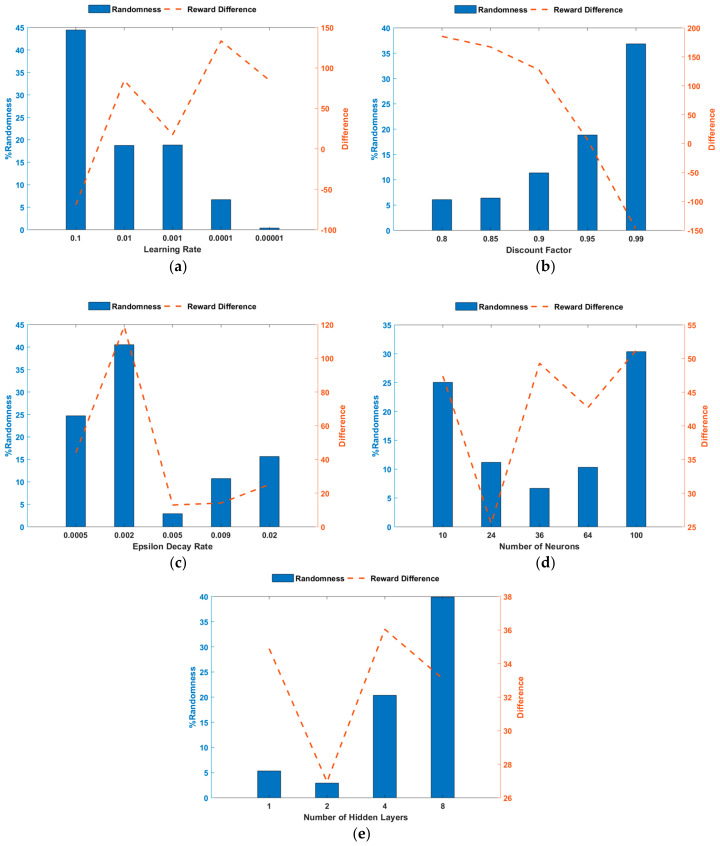
Q0 analysis concerning change in (**a**) the learning rate; (**b**) the discount factor; (**c**) the epsilon decay rate; (**d**) the number of neurons in each layer of the NN; and (**e**) the number of hidden layers of the NN.

To further assess the training performance and increase the reliability of the developed ORPD-DQN-based agent, the critic estimate of future rewards (Q0) for each of the examined hyperparameters is evaluated. Q0 is the estimate of the discounted long-term reward at the start of each episode, with the initial observation of the environment. It indicates the suitability of the modeled NN for the defined problem. A well-designed critic is implied by the Q0 approaching the true discounted long-term reward as training progresses. Thus, analytically, the value of Q0 should be numerically in the same range as the episode reward. In this paper, Q0 is examined through observing two parameters: (1) $R_{Q0}$, randomness throughout training, which also covers the occurrence of spikes, and (2) $D_{R\_max}$, the difference with respect to the episode reward at $E_{R\_max}$. Based on the considered network, these parameters are analyzed for the different values of the studied hyperparameters. Figure 6 presents the Q0-related indices, $R_{Q0}$ and $D_{R\_max}$, observed trends for the RL-agent and NN hyperparameters, respectively. The following can be realized from the Q0-related analysis:

- An LR of 0.1 shows the lowest performance since it has the highest changes in the critic estimate, implying that it does not show a specific trend and has several spikes. This can also be deduced from the negative $D_{R\_max}$,
- LRs of 0.01, 0.001, and 0.0001 exhibit better training performances where the Q0 value increases to a value near the episode reward and lower arbitrariness. Although an LR of 0.0001 shows enhanced results in terms of randomness, the final Q0 value is far from the episode reward, and similar to a 0.0001 LR performance, an LR of 0.00001 implies that the final Q0 does not approach the true discounted long-term reward.
- A DF of 0.95 shows adequate performance in terms of both $R_{Q0}$ and $D_{R\_max}$ compared to the other values. It can be concluded that generally, as DF increases, the training encompasses higher randomness. However, the final value of Q0 becomes closer to the true one. Nevertheless, if the value is increased beyond the most suitable value, the critic estimate exceeds the true value.
- An EDR of value 0.002 for the defined ORPD problem exhibits the weakest performance, and other values present comparable values in terms of $R_{Q0}$ and $D_{R\_max}$, with an EDR of 0.005 displaying improved training performance.
- By neighboring the relevant value of the number of neurons and hidden layers in the NN for the defined problem, steadier and more enhanced Q0 behavior is observed.

#### 4.1.3. The ORPD DQN-based Hyperparameters

Several challenges are confronted by implementing DRL to solve the ORPD problem. One of the major challenges is tuning the hyperparameters and deciding the optimal combination. This task can be extensively time-consuming since each parameter impacts the agent’s performance differently. Also, due to the wide choice of parameters, examining all possible sets of hyperparameter combinations via trial and error translates to a costly number of trainings. Thus, the large number of factors affecting the performance increases the complexity of this task and makes the optimal combination of hyperparameters case-specific. For the ORPD problem defined in this paper and the analysis performed for the different hyperparameters with respect to the defined performance indices, the optimal combination of hyperparameters exhibited superior performance of the ORPD DQN-based agent is provided in Table 3.

### 4.2. Reward Representation

**Figure 7:**
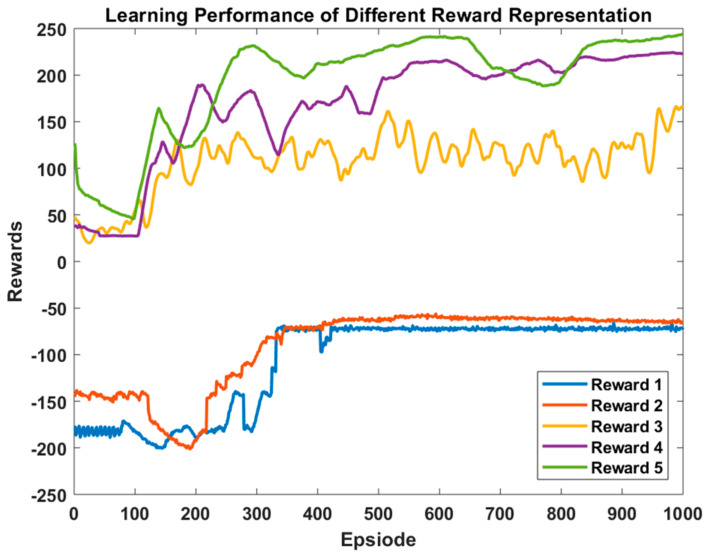
The learning performance of ORPD DQN-based agent for different reward representations.

Besides the hyperparameters configuration, the formulation of the reward function significantly impacts the agent’s learning performance and is considered a major task in developing RL algorithms. Due to the sensitivity of the learning performance of the DRL agent towards the reward and penalty representations, we hereby analyze and compare the learning performance of adopting several total reward function representations. The penalty formulations $r_{2}$, $r_{3}$, and $r_{4}$ in (5), (6), and (7), respectively, are fixed, and the reward formulation $r_{1}$ is varied. The total reward representation is then calculated as in (8). For the analysis of the reward representations, the ORPD hyperparameters in Table 3 are employed, and the value of the constants N, M, and G are adjusted for each representation. The studied reward representations are presented in Table 4, and the DRL agent learning performance concerning each reward formulation is plotted in Figure 7.

While Reward 1 is the simplest mathematically formulated and most used in previous work, it is observed that it results in one of the worst performing learning processes of the agent. Its convergence time is slow and has a greater likelihood of getting stuck in suboptimal solutions and, thus, does not reach the optimal policy for controlling the reactive output power of the PV. On the other hand, the squaring reward function (Reward 2) exhibits a slightly better performance in terms of convergence time and minimizing power loss by 10% and 2%, respectively. Nevertheless, the agent does not achieve the optimal policy. Reward 3’s formulation is mathematically more complex. However, the inclusion of the previous experiences results in a better power loss optimization than Reward 2 by 5%. Nonetheless, the agent training shows less stability with unstable progression of obtained rewards as training elapses. Differently, taking the reciprocal of power loss (Reward 4) enhances the stability of the agent’s training and exhibits a more robust learning performance. Furthermore, it significantly bolsters the learnt policy and shows a marginally improved convergence time. The reward formulation in Reward 5 outperforms the other reward representations, indicated via a more robust learnt policy and improvement in power loss minimization and convergence time. Compared to the most adopted representation, Reward 1, formulating the reward by taking its complement prompted the power loss minimization by 10–15% and shows a 14–18% improvement in convergence time. The reward representation analysis showcases the impact of formulating the reward function on the agent’s learning performance in terms of convergence time and learnt policy.

### 4.3. Scalability

In addition to several hyperparameters and reward representations impacting the agent’s convergence and training time, other factors such as the size of the training dataset and the state and action representations influence the training performance. Thus, it is crucial to investigate the impact of increasing the state and action spaces on computational time. In this paper, only 1 PV is utilized in the ORPD problem. However, as the number of assets—other distributed energy resources or CBs—increases, the action space size also increases, which translates to more training time. Thus, it might require more computing resources. Accordingly, the impact of scalability is studied in this subsection. This is performed through incorporating more assets, such as distributed energy resources or capacitor banks. 

For the studied cases, the hyperparameters in Table 3 are adopted, and the total reward representation in (8) is followed. The model is trained for a total of 5000 episodes on the CPU, using an i7-8550U, 16 GB RAM at 1.8 GHz CPU computing resource. Table 5 illustrates the approximate training time for different state and action spaces for the ORPD problem. It can be realized that with the increase in state or action spaces sizes’, the agent necessitates more time to finish the 5000 episodes. In other words, scaling the RL problem comes at the cost of training time and computing resources.

### 4.4. DQN-ORPD Performance Evaluation

To evaluate the proposed approach for parameter fine-tuning of the DRL algorithms, the active power loss minimization and voltage deviation improvement are assessed and compared with other approaches. The examined voltage deviation is of the voltage profile from the reference voltage 1.0 p.u. The base case is used as a benchmark and corresponds to the IEEE 33-bus system without any optimization. The proposed DQN-ORPD approach is compared with recent works aimed at solving the ORPD. The work in paper [56] applied mixed integer convex nonlinear programming (MIC-NLP) methodology, while in paper [57], the salp swarm algorithm (SSA) was employed to minimize active power loss. In addition, the genetic algorithm (GA) and the soft-actor-critic RL algorithm are investigated. Table 6 highlights the percentages of reduction in power loss and improvement in bus voltages compared to the base case. The hyperparameters tuning of the DQN-ORPD achieves additional power loss reductions, as well as better enhancements of voltage profiles compared to other tested approaches and recent works.

## 5. Conclusions

Recently, DRL has been successfully employed for power system control and optimization problems in place of other machine learning and conventional approaches. Several challenges are faced when implementing DRL. This paper examines the impact of several RL elements on the agent’s learning performance by solving the ORPD problem using DQN. The main elements that impact the RL performance are identified as follows: reward function representations, training hyperparameters, and action and state space sizes. The studied hyperparameters include the learning rate, the discount factor, the epsilon decay rate, the number of hidden layers in the critic network, and the number of nodes in each hidden layer. The training performance of the agent is assessed based on multiple performance indices from the episode and critic estimate curves. It is observed that adequate agent performance is observed to be neighboring the best-suited value of each hyperparameter for the studied problem.

In addition, different reward function formulations are examined for the ORPD case study. Some representations outperform others in terms of robustness, learnt policy, and convergence speed. Results show that the complementary reward function exhibits, in comparison to other representations, improved performance in terms of power loss minimization and convergence time by 10–15% and 14–18%, respectively. Furthermore, we investigated the scalability and computing resources required. To be able to incorporate a large number of assets, i.e., increase the state and action spaces, higher computing resources are required for adequate training time. Scalability analysis depicts that increasing number of possible action combinations in the action space by approximately nine times, results in 1.7 times increase in the training time. Furthermore, this paper provides a set of hyperparameters that can serve as a useful starting point for similar problems and an inspiration for new problems.

There are several potential directions for future research. Firstly, expanding the ORPD problem formulation to a multi-objective problem and investigate the impact of the DRL performance sensitivity elements. Secondly, considering a multi-agent DRL approach and examine the hyperparameters tuning methodology.

## Figures and Tables

**Table 1:** Recent studies analyze RL agent performance with respect to different evaluation metrics, hyperparameters, reward formulations, and scalability in different domains (LR: learning rate, DF: discount factor, EDR: epsilon decay rate, NN: neural network).

Ref	Evaluation Metric			Hyperparameters				Optimal Configuration	Reward	Scalability
	Accuracy	Time	Critic Estimate	LR	DF	EDR	NN			
[48,49]	✗	✗	✗	✗	✗	✗	✗	✗	✔	✗
[50]	✔	✔	✗	✗	✗	✗	✗	✗	✔	✔
[51]	✔	✔	✗	✔	✗	✗	✗	✗	✗	✗
[52]	✔	✗	✗	✗	✔	✗	✔	✔	✗	✔
This paper	✔	✔	✔	✔	✔	✔	✔	✔	✔	✔

**Table 2:** System Parameters.

Parameter	Value
Peak demand	8.0 + 2.6 MVA
Substation voltage	1.04 p.u (12.67 kV)
$V_{min}$	0.95 p.u
$V_{max}$	1.05 p.u
$S_{max}{}^{PV}$	1.5 MVA

**Table 3:** ORPD hyperparameters configuration.

Parameter	Value
Learning rate	0.001
Discount factor	0.95
Epsilon decay rate	0.005
Number of nodes	24
Number of hidden layers	2

**Table 4:** The learning performance of ORPD DQN-based agent for different reward representations.

Reward Description	Reward Representation
Reward 1: negative power loss	$r_{1}=-p$
Reward 2: negative power loss squared	$r_{1}=-p^{2}$
Reward 3: delta power loss	$r_{1}=p_{t-1}-p_{t}$
Reward 4: reciprocal of power loss	$r_{1}=1/p$
Reward 5: complement of power loss	$r_{1}=1-p$

**Table 5:** Training time in seconds for the different number of hidden layers in the critic network.

ORPD Utilized Assets (Action Space)	Approximate Training Time for 5000 Episodes (Days)
3 capacitor banks, each with ON/OFF actions	3.2
3 capacitor banks, each with ON/OFF actions + 1 PV with an inverter rating of 0.6 MVA (step size 0.1)	5.1
3 capacitor banks, each with 4 possible actions + 1 PV with an inverter rating of 0.6 MVA (step size 0.1)	9.4

**Table 6:** Comparison between different approaches for solving the ORPD problem on the IEEE 33-bus system in terms of power loss and voltage deviation, considering the base case (no optimization) as reference.

Approach	% Power Loss Minimization	% Voltage Deviation Improvement
MIC-NLP [56]	17.15	Not mentioned
SSA [57]	17.12	Not mentioned
GA	17.24	11.40
SAC	18.11	11.92
DQN	18.89	12.08
